# The Occurrence, Biosynthesis, and Molecular Structure of Proanthocyanidins and Their Effects on Legume Forage Protein Precipitation, Digestion and Absorption in the Ruminant Digestive Tract

**DOI:** 10.3390/ijms18051105

**Published:** 2017-05-22

**Authors:** Arjan Jonker, Peiqiang Yu

**Affiliations:** 1 Department of Animal and Poultry Science, College of Agriculture and Bioresources, University of Saskatchewan, 51 Campus Drive, Saskatoon, SK S7N 5A8, Canada; 2 Grasslands Research Centre, AgResearch Ltd., Tennent Drive, Private Bag 11008, Palmerston North 4442, New Zealand

**Keywords:** proanthocyanidins, condensed tannins, flavonoid pathway, biosynthesis, molecular structure, rumen and intestinal protein metabolism and adsorption

## Abstract

Forages grown in temperate regions, such as alfalfa (*Medicago sativa* L.) and white clover (*Trefolium repens* L.), typically have a high nutritional value when fed to ruminants. Their high protein content and degradation rate result, however, in poor utilization of protein from the forage resulting in excessive excretion of nitrogen into the environment by the animal. Proanthocyanindins (also known as condensed tannins) found in some forage legumes such as birdsfoot trefoil (*Lotus corniculatus* L.), bind to dietary protein and can improve protein utilization in the animal. This review will focus on (1) the occurrence of proanthocyanidins; (2) biosynthesis and structure of proanthocyanidins; (3) effects of proanthocyanidins on protein metabolism; (4) protein precipitating capacity of proanthocyanidins and their effects on true intestinal protein adsorption by ruminants; and (5) effect on animal health, animal performance and environmental emissions.

## 1. General Introduction

Forages, such as alfalfa, white clover (*Trifolium repens* L.) and perennial ryegrass (*Lolium perenne* L.) are the major forages used in temperate regions because of their high yield and nutritive value. They are, however, characterized by having a high protein content which is excessively degraded in the rumen, resulting in poor protein use efficiency and excessive nitrogen excretion into the environment [1]. Proanthocyanidins, which are present at moderate levels in temperate/prairie forages such sainfoin (*Onobrychis viciifolia* L.), birdsfoot trefoil (*Lotus corniculatus* L.), big trefoil (*Lotus pendunculatus* L.) and sulla (*Hedysarium coronarium* L.) bind with dietary proteins in the rumen, which can improve protein utilization in the ruminant animal. Of note, the beneficial effects of proanthocyanindin described in this manuscript are relevant to forages with high protein concentrations (approximately over 18% of feed dry matter (DM), but proanthocyanidin may not be, or less, beneficial in forages and diets with adequate (12–18%) or low protein concentration relative to animal requirements.

## 2. Proanthocyanidin Synthesis and Structure

**Figure 1:**
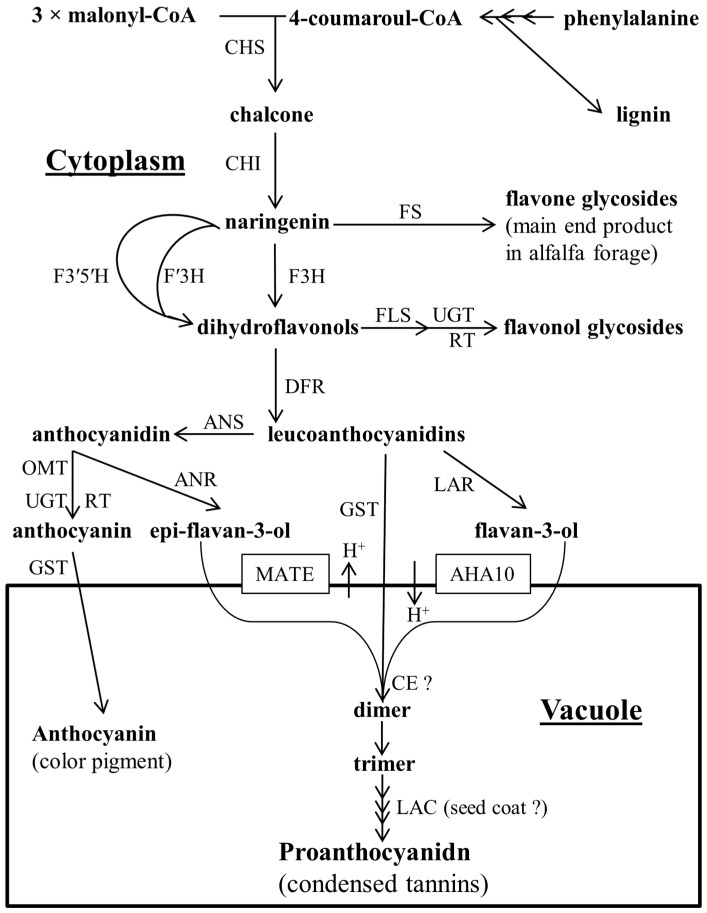
Biosynthetic pathway for anthocyanin and proanthocyanidin. Abbreviations for enzymes involved in the flavonoid pathway towards the synthesis of proanthocyanidin are as follows. CHS: chalcone synthase; CHI: chalcone isomerase; FS: flavone synthase; F3H: flavanone-3-hydroxylase; F′3H: flavonoid 3′ hydroxylase; F3′5′H: flavonoid 3′5′ hydroxylase; FLS: flavonoid synthase; UGT: UDP-dependent glucosyltransferase; RT: rhamnosyl transferase; DFR: dihydroflavonol 4-reductase; ANS: anthocyanidin synthase; ANR: anthocyanidin reductase; LAR: leucoanthocyanidin reductase; OMT: *O*-methyltransferase; GST: glutathione S transferase; MATE: multidrug and toxic compound extrusion-type transporter; AHA10: plasma membrane H^+^-ATPase; CE: condensing enzyme; and LAC: laccase-like flavonoid oxidase, ?: unknown. This figure was prepared with information obtained from Kleindt et al. [12] and Zhao et al. [13].

**Figure 2:**
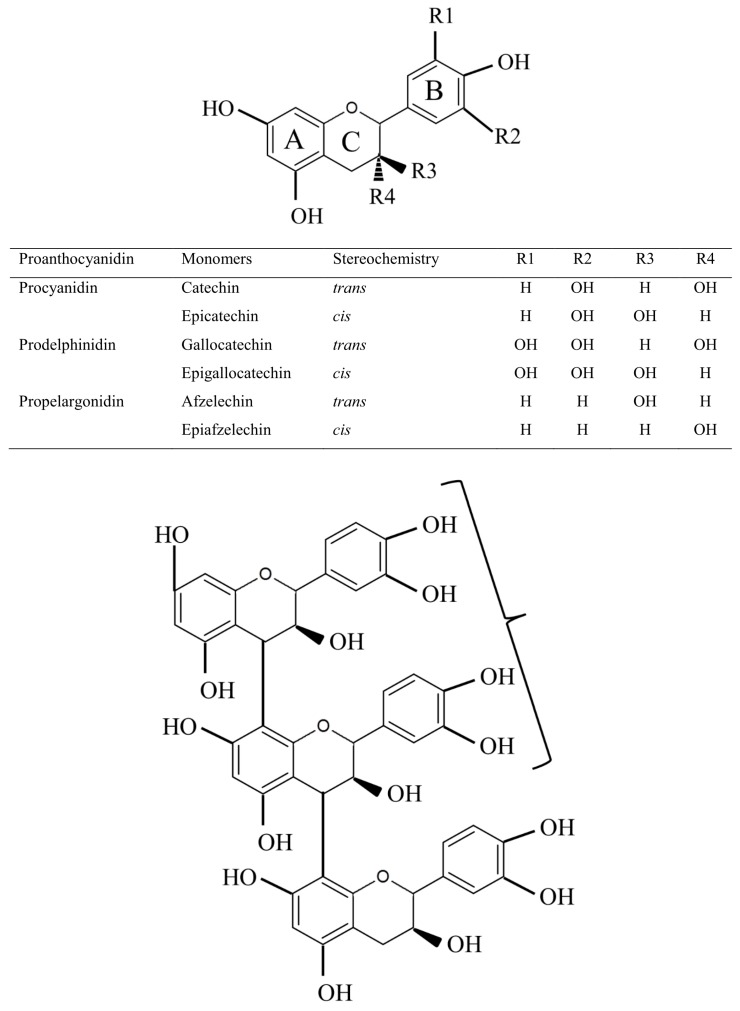
Structure of (epi)-flavan-3-ol and substitution patterns of proanthocyanidins found in legumes. This figure was prepared with information obtained from Marles et al. [2].

Proanthocyanidins are oligomeric and polymeric linked flavonoid units synthesized in the flavonoid pathway. The name proanthocyanidin comes from the red anthocyanidin formed after polymer cleavage and acidic oxidation upon heating [2]. Monomeric flavonoids are synthesized in the cytosol of the plant and are subsequently transported into the vacuole to form end-products like proanthocyanidins and anthocyanins [3]. Proanthocyanidins are synthesised in the flavonoid pathway, which starts with the condensation and subsequent cyclization of one molecule of 4-coumaroyl CoA (synthesised in the phenylpropanoid pathway from phenylalanine via cinnamic acid and coumaric acid) and three molecules of malonyl CoA (formed by carboxylation of acetyl CoA) to form chalcone (Figure 1). Flavonoids, starting with chalcone, contain a 15-carbon backbone (C15) in a C6-C3-C6 skeleton, which contains two phenyl rings (an A ring, originating from 3× malonyl CoA cyclization and a B ring, originating from phenylalanine) (Figure 2). These two rings are connected by a three-carbon bridge to form a third ring (C3 ring) by isomerization in the next step of the pathway towards naringenin. Dihydroflavonols and leucoanthocyanidin are formed in the next two steps of the pathway by hydroxylation of the C3 ring and reduction of the C4 C ring, respectively [2,4,5]. The building blocks of proanthocyanidins are flavan-3,4-diols (leucoanthocyanidins) which form a dimer with either flavan-3-ols (e.g., (+)-catechin, (+)-gallocatechin and (+)-afzelechin) [4,6] or epi-flavan-3-ols (e.g., (−)-epi-catechin, (−)-epi-gallocatechin and (−)-epi-afzelechin) (Figure 2). Anthocyanidins (e.g., delphinidin and cyanidin) are the precursors for both epi-flavan-3-ols and anthocyanin [2,7]. Proanthocyanidin can be characterized in terms of total concentration of extractable and unextractable fractions (sometimes further divided into protein- and fibre-bound) [8], molecular size in terms of degree of polymerization (mDP, total flavanol units/terminal flavanol units) or molecular weight (MW), prodelphinidin/procyanidin ratio (PD/PC; (galocatechin + epi-galocatechin)/(catechin + epi-categin)), *cis*/*trans* ratio (orientation at C-ring; (epi-catechin + epi-galocatechin)/(categin + galocategin)) [9], using protein precipitation capacity (PCC) assay [10] and in vitro or in vivo bio-assay with and without polyethylene glycol (PEG) to deactivate the activity of proanthocyanidin [11]. 

## 3. Occurrence of Proanthocyanidin in Temperate/Prairie Forages

Proanthocyanidins are typically found in the leaves, stems, flowers and seeds of forage legumes [9,14]. Some forage legumes like sainfoin and birdsfoot trefoil contain proanthocyanidins in all parts of the plant [14], while in alfalfa, perennial ryegrass and tall fescue (*Festuca arundinacea*) they accumulate mainly in the seed coat [15,16] and in white clover and red clover (*Trefolium pratense* L.) mainly in the flowers [17,18]. However, trace concentrations of proanthocyanidin were detectable in areal parts of most temperate forages [19,20]. 

In sainfoin leaves, proanthocyanidin concentrations were higher, with a higher mDP and higher prodelphinidin content (Figure 2), than in the stems [21,22]. During sainfoin leaf development, proanthocyanidin concentration, MW and mDP increase until the leaves start to unfold, after which the concentration of these compounds decreases until senescence [23,24]. Sainfoin proanthocyanidin concentration and structure were also affected by growth site, harvest number and single vs. multiple flowering types [25,26]. Sulla was found to have about seven times greater proanthocyanidin concentrations in both leaves and flowers than in stems [27], while purple prairie clover had greater proanthocyanidin concentrations in flowers than in leaves, which both had much greater concentrations than stems [14,28]. Unlike for sainfoin, proanthocyanidin content was higher at more advanced states of maturity in forage of birdsfoot trefoil, purple prairie clover (*Dalea pupurea* L.) and several *Trifolium* species [29]. 

In addition to the stage of growth of the plant, the proanthocyanidin concentration is influenced by the environmental conditions under which the plant is grown. Big trefoil accumulated more proanthocyanidins when grown at 30 °C than at 20 °C in a growth cabinet [30]. The proanthocyanidin content and PPC were higher in temperate and tropical legumes grown in low fertility soils compared with high fertility soils [31,32,33] and were higher in the dry season than the wet season for a tropical legume forage grown in Columbia [34]. In the western Canadian prairies, growing season did not affect the proanthocyanidin content of temperate legumes [29]. Herbivores and insects foraging on proanthocyanidin-containing plants caused wound-induced up-regulation of the flavonoid pathway regulatory genes with concomitant increases in proanthocyanidin accumulation in aspen trees (*Populus termuloides* Michx.) [35] and turtlegrass (*Thalassia testudinum* L.) [36]. 

## 4. Proanthocyanidin Release from the Plant

**Figure 3:**
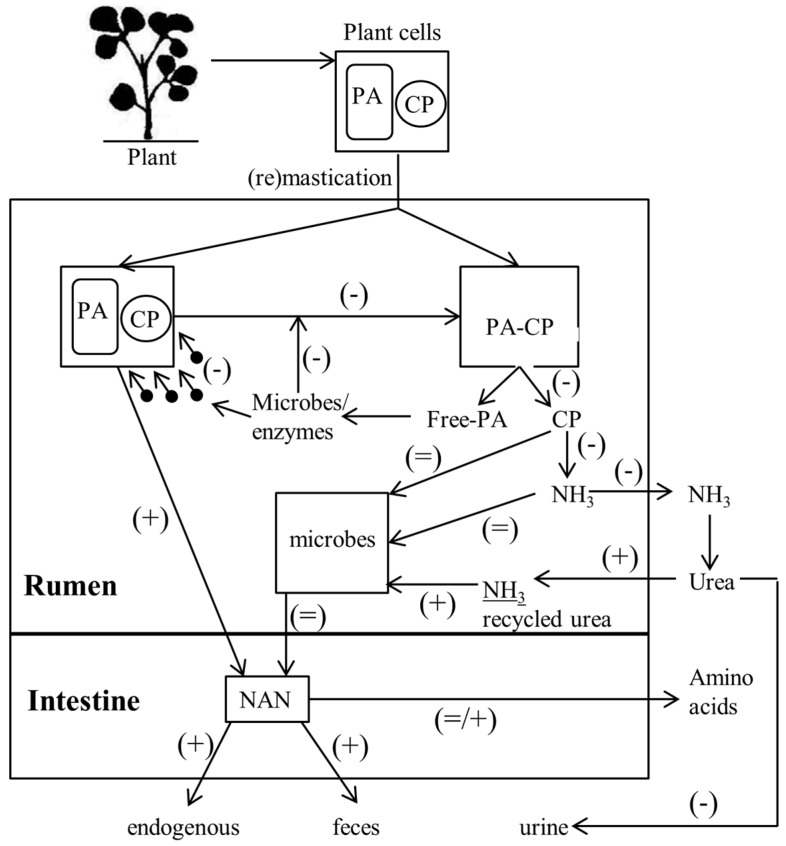
Schematic flow chart of crude protein (CP) digestion from proanthocyanidin (PA)-containing forage. Symbols between brackets represent the effect of PA-containing vs. PA-free forage on protein flow: + represents increased flow, − represents decreased flow and = represents similar flow. NAN: non-ammonia N. This figure was prepared with information from references [38,39,45,46,47].

Proanthocyanidins are stored in the vacuole of plants in order to prevent interaction with any enzymes involved in the metabolic processes of the plant. In forage legumes, the vacuoles which contain proanthocyanidin are more abundant under the adaxial epidermis extending into the mesophyll and more frequently around the stomata [21,37]. Microbes attach rapidly to any new feed that enters the rumen. When proanthocyanidins are present in the plant cell, attachment of microbes to the plant/feed tissue is much slower, which decreases the invasion of plant tissues (cells) by microbes (Figure 3) [38]. Therefore, plant tissues which contain proanthocyanidins are ruptured more slowly and less extensively than plant tissues that do not contain proanthocyanidins. This reduces the accessibility of the cell contents and fibre components for microbial utilization [38,39].

When the vacuole is ruptured through chewing or microbial digestion, proanthocyanidins can bind with surrounding proteins (mainly proteins from within the plant tissue), but also dietary, salivary and microbial protein (Figure 3). The proanthocyanidin–protein complex is very resistant to digestion and utilization by ruminal microbes [40,41].

During ingestive chewing in sheep, large amounts of soluble protein were released (ruptured) from proanthocyanidin-free forages like alfalfa, perennial ryegrass and red clover, but not from proanthocyanidin-accumulating forages like sainfoin. However, when PEG was added, approximately 60% of the soluble protein in sainfoin forage was released, indicating that the proanthocyanidins in sainfoin forage were responsible for the lower release of soluble proteins compared with the other forages tested [42]. Similar results were found in vitro in buffer, where adding PEG increased nitrogen (N) solubility four-fold in fresh sainfoin forage, while PEG had no effect on the N solubility of alfalfa [43]. Theodoridou et al. [9] also found increased N solubility of fresh sainfoin forage with PEG addition and the magnitude in response to PEG was related to proanthocyanidin concentration. A negative correlation was found for N solubility and protanthocyanindin concentration, PD/PC ratio, mDP and *cis*/*trans* ratio for three sainfoin varieties at several harvests [44]. 

## 5. Protein Precipitating Capacity as Affected by Proanthocyanidin Characteristics 

Protein precipitation by proanthocyanidins is mostly based on hydrogen bonding between the hydroxyl groups (–OH) of proanthocyanidin and the amino group (–NH) of peptides or other substrates [48] or is due to hydrophobic interactions between the phenol ring of proanthocyanidin and the carboxyl group (–COOH) of protein. Ionic interaction and covalent bonding occurs less frequently [3]. Proanthocyanidins can also bind to metals, essential amino acids, carbohydrates, digestive enzymes, and microbes, but with a lower affinity than that used to bind to dietary protein [49,50]. The composition of proanthocyanidin varies with the type of linkage between the flavonoid monomers (C4 to C6 or C4 to C8) and with stereochemical variation at carbons 2, 3 and 4 and the number of hydroxyl groups on the A and the B rings (Figure 2). These differences in proanthocyanidin composition affect their molecular structure and influence their capability to interact with other molecules such as protein. 

Factors that were found to increase PCC of proanthocyanidins include increasing proanthocyanidin concentration, increasing MW, larger mDP and increasing prodelphinidin:procyanidin ratio [51,52,53,54]. Nauman et al. [55] found that proanthocyanidin concentration, determined in nine warm season forages, correlated with PCC, while mDP (and MW) did not. Also, Huang et al. [56] found no clear effect of mDP on PCC and suggested that proanthocyanidin chemical structure could provide a better understanding of PPC. Aerts et al. [57] suggested based on their results that the MW of proanthocyanidin was relatively more important for PCC while monomer composition of proanthocyanidin was relatively more important in determining their interaction with microbes. Ropiak et al. [58] found that mDP of proanthocyanidin was the most important factor determining PCC, while monomer composition of proanthocyanidin was more important in explaining the interaction of proanthocyandin with protein in terms of hydrophobic binding and changing protein secondary structure. Several studies compared the PCC of proanthocyanidin fractions of different MW and all found that PCC increased with increasing mDP [17,59,60,61]. Habertson et al. [62], however, suggested that mDP above eight would not further improve PCC effectiveness. Molan et al. [63] found that in vitro degradation of small and large subunits of Rubisco (Ribulose-1,5-bisphosphate carboxylase) decreased linearly when incubated with dimers to pentamers of procyanidin, but this was similar for the incubation with pentamers and purified birdsfoot trefoil proanthocyanidin (dominated by procyanidin). Ropiak et al. [58] found that proanthocyanidin with an mDP of 7 had optimal PCC with bovine serum albumin (BSA) based on a wide range of purified proanthocyanidins. 

Jones et al. [52] found that PPC increased with increasing prodelphidin content when testing several *Trifolium* species and big trefoil. Prodelphinidins have three phenolics at the B-ring compared to two in procyanidin, which may explain [20] a higher PCC of proanthocyanidin with high PD/PC ratios [64]. The PD/PC ratio was higher in fractions with higher mDP from proanthocyanidin of big trefoil [65,66], birdsfoot trefoil [66], sainfoin [67] and to a lesser extent in sulla [68] and *Dorycium rectum* [69] (Table 1). Ropiak et al. [58] did find, however, using a PCC with bovine serum albumin (BSA) and a wide range of different types of purified proanhocyanidin with PD/PC ratios ranging from 0/100 to 99:1, *cis*/*trans* ratios from 1/99 to 88/12 and MW from 1028 to 7580 Da, that MW and mDP were the main parameters explaining PCC, while PD/PC ratio and *cis*/*trans* ratio did not correlate with PCC. A similar result was found by Lorenz et al. [70] using purified proanthocyanidin from sainfoin with a wide range of PD/PC and *cis*/*trans* ratios. However, interaction between proanthocyanidin and protein in terms of hydrophobic binding and secondary structure determined by tryptophan fluorescence quenching and circular dichroism, respectively, were mainly influenced by procyanidin and prodephinidin content of the proanthocyanidin, respectively [58]. 

The PCC with Rubisco was similar among proanthocyanidin from birdsfoot trefoil, big trefoil, sainfoin and sulla with between 25 and 75 µg of proanthocyanidin extract required to precipitate 10 µg Rubisco [19,54]. Rate of degradation of the rubisco large sub-unit was also similarly reduced by purified proanthocyanidin from birdsfoot trefoil, big trefoil, sainfoin and sulla [40]. A negative correlation was, however, found for fractional degradation rate of protein in the rumen and effective ruminal protein degradability with increasing protanthocyanindin concentration, mDP, PD/PC ratio and *cis*/*trans* ratio for three sainfoin varieties at several harvests [44]. Proanthocyanidin of leaves from sainfoin were found to have a stronger PCC with BSA than for proanthocyanidin from stems, likely due to differences in mDP of proanthocyanidin in leaves and stems [9]. Proanthocyanidin from purple prairie clover were found to have a stronger PCC than of sainfoin [71] and PCC was weak for temperate forages that contain trace concentrations of proanthocyanidin [19,54]. In summary, concentration and mDP (or MW) appear as the main proanhocyanaidin characteristics that determine their PCC with dietary protein.

## 6. Protein Precipitating Capacity of Proanthocyanidins as Affected by Protein Characteristics 

The PCC of proanthocyanidin depends not only on the structure but also on characteristics of protein they bind to [17,70]. Protein precipitating capacity was found to be weaker for BSA than for alfalfa leaf protein (Rubisco) [17,54,70], rapeseed protein [70], and proline-rich protein (gelatine) [58], but similar to the PCC with the enzyme lysozyme [17]. Results from Lorenz et al. [70] suggested that high over low MW proteins were preferentially precipitated, however, this was more apparent with rapeseed than Rubisco protein. The degradation of the large subunit of Rubisco in the rumen is normally more rapid than the ruminal degradation of the small subunit of Rubisco [40,41]. The presence of proanthocyanidins from sainfoin decreased the degradation of the large sub-unit of Rubisco more than it affected the degradation of the small sub-unit of Rubisco [40,41]. However, proanthocyanidins from sulla and big trefoil did not differ in their ability to reduce microbial degradation of the small or large sub-unit of Rubisco [57]. This suggests that proanthocyanidins from different forage legumes differ in their biological activity in ruminal Rubisco degradation. The quaternary structure of Rubisco is relatively unstable compared to the quaternary structure of BSA, which might explain why Rubisco is more readily precipitated by proanthocyanidin than BSA [17]. Proanthocyanidin might also more readily bind with a mix of proteins, as is the case of Rubisco and rape seed protein, than with individual proteins as for BSA [17,70]. Proteins with great proline content, such as gelatine, contain randomly coiled structures which offer more binding sites for proanthocyanidin than is the case for BSA [58]. The protein secondary structures of BSA was found to change during the PCC assay with relative decreasing α-helices and increasing β-sheets as the prodelphinidin content of the proanthocyanidin increased [58]. This suggests that protein with high α-helices might be more easily precipitated, although the protein of alfalfa (consisting mainly of Rubisco) had a lower α-helices: β-sheets ratio [78,79] than BSA [58].

## 7. Effect of Proanthocyanidin on Rumen Microbes and Ammonia Formation

Excess protein released in the rumen above microbial requirement is mainly converted into ammonia (NH_3_), and energy for the microbes, which is absorbed through the rumen wall and largely excreted in urine as urea [1]. Reducing the degradation rate and extent of protein in the rumen can decrease NH_3_ formation and urinary N excretion [1]. Sheep fed birdsfoot trefoil or big trefoil forage had a lower ruminal NH_3_ and soluble protein concentration than sheep fed the same diet plus PEG [45,46,80]. A linear decrease in rumen NH_3_ was found with increasing concentration proanthocyanidin in the diet in a review of studies with animals fed temperate legumes [74] and a meta-analysis of data from animals fed a wide range of proanthocyanidin sources [81]. These could be the result of reduced dietary protein availability due to complexing with proanthocyanidin (as described above), negative correlation between forage crude protein (CP) concentration and proanthocyanidin concentration (reduces the direct oversupply of protein) [82,83,84,85], or overall reduced proteolytic activity due to the direct effect of proanthocyanidin on proteolytic bacteria and protozoa in the rumen [80] (Figure 3). 

Proanthocyanidins that do not bind with protein are referred to as free proanthocyanidins. During ingestive mastication of birdsfood trefoil and sulla (*Hedysarium cornonarium* L.) by sheep, 14 and 21 g/kg DM of extractable proanthocyanidins were converted into 11 and 12 g/kg DM protein-bound and 3 and 6 g/kg DM protein-free proanthocyanidins, respectively [86,87]. The proanthocyanidins that bind to protein are considered to be beneficial for the animal because they increase the protein flow to the lower digestive tract [88,89] while free proanthocyanidins are considered negative because they negatively affect fibre digestion, especially the digestion of hemicellulose [88,90]. The proanthocyanidins which were still extractable after ingestive mastication were probably located in plant cells that were not yet ruptured. For birdsfoot trefoil, 17% of the original extractable proanthocyanidin was still present after ingestive mastication in contrast to the 31% observed for sulla [86,87], which might indicate that the plant tissue from birdsfoot trefoil is more easily ruptured than that from sulla.

Free proanthocyanidins can interact directly with minerals, microbes and microbial enzymes and reduce the overall proteolytic activity (protein degradation) in the rumen [19,40,80,91,92]. Proanthocyanidins inhibit fibrolytic, ureolytic and proteolytic enzyme activity by microbes and thereby inhibit the growth of fungi, protozoa and some bacteria species [80,91,92]. Some proteolytic bacteria species are affected by proanthocyanidins, while other species seem unaffected [80,92,93]. For example, proanthocyanidin promoted the growth of proanthocyanidin-resistant gram-negative bacteria in the rat gastrointestinal tract. Proanthocyanidin resistant microbes increased from <1% before feeding proanthocyanidin in the diet to approximately 25% and 50% proanthocyanidin resistant microbes after three weeks of offering a feed containing 0.7% and 2.0% proanthocyanindin, respectively [94]. Microbial growth in the presence of proanthocyanidins might be decreased because of the reduced availability of essential nutrients (e.g., amino acids and minerals), reduced availability of total nutrients (e.g., carbohydrates and protein), complexes formed with microbial membrane lipoproteins, and direct interactions with the metabolism of microbial bodies [91]. Protozoa numbers are decreased by the presence of proanthocyanidins in the diet [95,96]. Protozoa increase the overall digestibility of organic matter, are highly proteolytic, degrade insoluble proteins, predate on bacteria (increasing ruminal-N turnover) and reside in the rumen for a longer period of time than bacteria [97,98]. However, the total flow of microbial-CP to the lower digestive tract is not decreased when proanthocyanidins are present in a forage (Figure 3) [45,80]. In the latter study, proathocyanidin-resistant microbial growth and/or reduced protozoa number improved microbial efficiency. Defaunation of protozoa from the rumen on its own was previously found to increase microbial protein flow to the lower digestive tract [97,98].

Sheep with a lower ruminal NH_3_ concentration have higher urea-N recycling and a higher incorporation of recycled urea-N into microbial mass (Figure 3) [45,99]. This might be an explanation of why the presence of proanthocyanidin in the diet does not decrease the overall flow of microbial-CP to the lower digestive tract. Decreased ruminal NH_3_ concentrations in cattle fed proanthocyanidin-containing forage decreased urinary-N output and increased faecal-N output [100,101]. Faecal-N is less prone to volatilization as ammonia and nitrous oxide and leaching as nitrate into ground water than urinary-N, thereby reducing the environmental impact of this N excretion by ruminants [101,102].

## 8. Effect of Proanthocyanidin on Intestinal Amino Acid Absorption

Proanthocyanidins form stable complexes with proteins from different sources at a pH between 3.5 and 7.0 [103], a pH which occurs in the rumen [104] and ileum [105]. The total amount of dietary protein escaping ruminal degradation into the lower digestive tract was found to be higher for proanthocyanidin-containing forage without PEG than in the presence of PEG [45,46,47]. Protein is released from the proanthocyanidin complex at a pH of <3 [103] which occurs in the abomasum [106,107] and proximal duodenum [105] and at a pH of >8 which occurs with pancreatic secretions [90]. Min et al. [74] found, in their review, a linear increase in non-ammonia N flow as proportion of N intake to the intestine with increasing proanthocyanidin concentration in the forage, while microbial N flow remained largely constant. 

The change in site of protein digestion due to dietary proanthocyanidin (compared to same feed + PEG) resulted in an increased digestion and absorption of amino acids in the small intestine of sheep eating birdsfoot trefoil [46,89] and sulla [47], but not when sheep consumed big trefoil [45] and sainfoin [47,108]. Kariuki and Norton [109] found that proanthocyanidin from *Leucaena leucocephala* L. had a lower PCC with BSA but this complex had a higher true digestibility between abomasum and distal ileum than when proanthocyanidin originated from *Leucaena pallida* L. The data in Table 1 indicates a higher protein PCC with BSA and proanthocyanidin from sainfoin and big trefoil than from birdsfoot trefoil. Based on the results of Kariuki and Norton [109], the lower PCC of proanthocyanidin from birdsfoot trefoil might result in a higher digestibility of protein, which was bound to proanthocyanidin between the abomasum and the distal ileum, than from sainfoin and big trefoil. This might be an explanation why the amino acid absorption in the small intestine increased (compared to same feed + PEG) when feeding birdsfoot trefoil and not when feeding sainfoin or big trefoil. Big trefoil was found to have a proanhocyanidin fraction with high mDP of 44 that was not detected in birdsfoot trefoil [66] which might explain difference in biological activity between the two *Lotus* species. Sulla was, however, also found to have a proanthocyanidin fraction with high mDP of 46 [68], like big trefoil. However, the particular proanthocyanidin fraction that had a high mDP was different for big trefoil and sulla. In vitro results by McNabb et al. [19] suggested that proanhocyanidin-rubisco complex of sainfoin was stable over a wider range of pH values than for birdsfoot trefoil and sulla, but also than for big trefoil. This suggests that the proanhocyanidin–rubisco complex might be less easily dissociated for sainfoin along the digestive tract. 

## 9. Effect of Proanthocyanidin on Intestinal Parasites

Parasitic nematodes are a major factor impairing animal growth in temperate grazing systems [110]. Feeding temperate legumes containing proanthocyanidin (e.g., birdsfoot trefoil, big trefoil, sulla, sainfoin) were found to decrease nematodes in vitro [111] and in vivo [112,113] in terms of reduced total counts, reduced numbers of eggs hatching and rate of larval development. The review of Min et al. [74] found a linear reduction in faecal egg counts with increasing proanthocyanidin concentration of different sources in the diet, with the effect being more apparent at proanthocyanidin concentrations of over 4.5% in the diet DM. This might, however, depend on feed and proanthocyanidin source. For example, reductions in faecal egg counts have been more consistent with sulla than with birdsfoot trefoil [112,113,114]. Several recent in vitro studies found that mDP and prodelphidinin content in proanthocyanidins were important factors determining anti-parasitic activity [115,116,117]. Klongsiriwet et al. [115] found that there was a synergistic effect of using proanthocyanidin and flavonoid monomers in increasing anti-parasitic activity, more so with procyanidin than with prodelphinidin. Grazed proanthocyanidin plants also contain monomerc flavonoids and might therefore be more effective against intestinal parasites than extracted fractions. Some caution is, however, required as Waghorn et al. [118] found that *Dorycnium rectum* was a very potent anti-parasitic agent in vitro, while the same forage grazed by sheep did not change anti-parasitic activity [119]. These authors therefore emphasized that in vitro anti-parasitic activity due to proanthocyanidin might not be a good indicator for in vivo activity. 

Indirect inhibition of intestinal parasites might also occur as a result of the improved protein supply to the small intestine with proanthocyanidin, which might improve the host immunity against parasites as reviewed previously [74,110]. 

## 10. Effect of Proanthocyanidin on Pasture Bloat

Pasture bloat arises from rumen microbial fermentation gases trapped in a viscous stable protein foam, that prevent normal eructation, causing distention of the rumen and thereby exerting pressure on organs which can lead to the death of the animal under severe conditions [1]. Many characteristic bloat-free legumes contain proanthocyanidins [120,121]. The proanthocyanidin–protein complex decreases the release of protein in the rumen. This reduces the amount of protein available at the gas–liquid interface [40,57] and decreases foam formation and stability [122,123,124] and substrate availability for ruminal microbes, with a consequent reduction in gas production [124,125]. Lysis of protozoa and gram-negative bacteria in the rumen release foam-provoking materials and exotoxins which may play a role in the formation of pasture bloat [126,127]. The numbers of protozoa and gram-negative bacteria are decreased by proanthocyanidins as described above. Also, the growth of the viscous slime-producing bacteria *Streptococus bovis* is impaired by the presence of proanthocyanidins [128]. According to Li et al. [129], bloat-provoking legumes should contain a proanthocyanidin concentration of approximately 0.5% of diet DM, or higher, in order to be bloat-safe. Mixing dock in a ratio of 1:9 with alfalfa, resulting in a dietary proanthocyanidin concentration of approximately 0.2% of DM, was sufficient to prevent bloat [130]. Proanthocyanidins in dock were found to have a strong PCC [19,130] and high proportion (27%) of epicatechin gallate [131] which are important antimicrobial properties [132]. 

## 11. Effect of Proanthocyanidin on Enteric Methane Emissions

Feeding forage that contained proanthocyanidins decreased methane emissions in sheep grazing sulla, birdsfoot trefoil and big trefoil [133,134,135] and in dairy cows grazing sulla and birdsfoot trefoil [136,137,138] compared with those grazing ryegrass-based pastures. Methane emissions were also reduced in goats fed *Sericea lespedeza* (*Lespedeza cuneate*) compared to goats fed alfalfa [139]. A meta-analysis indicated that methane emissions reduce linearly, both in vitro and in vivo, with increasing proanthocyanidin concertation (range of sources) [81]. The decreased methane emission with proanthocyanidin-containing forage might be due to a reduction in the amount of forage substrate fermented in the rumen (reduced digestion), a shift in fermentation end-products (reduced H^+^-producing acetate and to a lesser extent butyrate, and more H^+^-utilizing propionate and valerate), and/or direct inhibition of the growth of methanogenic bacteria, as well as a decrease in symbiotic-associated protozoa numbers or a shift in microbial community composition [81,140]. In vitro methane production and concentration were found to decrease with proanthocyanidin fractions of increasing MW and mDP from *Leucaena* [56] and with sainfoin ancestors with increasing mDP [141]. Methanogens were mainly inhibited with polymeric-proanthocyanidin fractions from big trefoil with a mDP of approximately 12 and not by oligomeric-pronthocyanidin fractions with mDP < 6 [142].

Methane emissions from beef cattle eating sainfoin were, however, in general not reduced compared to those eating alfalfa-based forage [143,144,145,146], except in one out of three grazing seasons [145,146]. Substituting 50% of grass silage with sainfoin silage in a total mixed ration for dairy cows was found to reduce methane yield [147]. Up to 2% quebracho proanthocyanidin mixed in the diet of beef cattle did also not lower methane emissions [148]. Proanthocyanidin of quebracho and sainfoind incubated without PEG decreased, however, methane production and concentration in vitro compared with incubations with PEG [9,141,149]. Therefore, not all proanthocyanidin sources may have the same effectiveness in reducing methane emissions. 

## 12. Absorption of Proanthocyandin and Health Benefits

Flavonoids from the lower part of the flavonoid pathway, including anthocyanidin and proanthocyanidin, have antimicrobial activity on pathogenic gram-negative bacteria [150], as well as strong anti-oxidant activity [50,151], anti-inflammatory activity [152] and the ability to change cell signalling pathways [153]. Livestock consuming these flavonoids might therefore experience beneficial effects important for the overall health of the animal. To experience these benefits at the metabolic level, however, proanthocyanidin needs to be broken down and absorbed into the blood stream. Available data suggests that proanthocyanidins are not broken down in the digestive tract of the ruminant and that nearly all proanthocyanidins ingested are excreted in faeces [53,117,154]. However, building blocks of proanthocyanidin present in all proanthocyanidin-accumulating forages are absorbed from the digestive tract. Plasma and urine of rats were found to contain monomeric flavonoids and dimmer and trimer procyanidin [155,156] and even up to pentamers of apple procyanidin [157]. In cows, however, ruminal administration of green tea flavan-3-ols did not result in a rise of flavan-3-ols in plasma, while post-ruminal administration did increase plasma flavan-3-ols in a dose-dependent manner [158]. Green tea flavan-3-ols appeared to be extensively metabolized in the rumen, which was confirmed in vitro [158]. Di Trana et al. [159] found, however, a positive correlation between proanthocyanidin intake and plasma antioxidant capacity, and plasma total polyphenol and milk total polyphenol concentrations in dairy goat fed fresh sulla. Supplementation of quebracho proanthocyandin in the diet of sheep also enhanced plasma and liver antioxidant capacity, however, no phenolic compounds were detected in plasma, which suggests that none of the quebracho proanthocyandin were absorbed from the digestive tract [160]. These authors discussed how proanthocyanidin as an antioxidant in the digestive tract might improve overall animal antioxidant status. Huang et al. [161] found an improved antioxidant status in serum of sheep fed purple prairie clover compared with sheep fed alfalfa. The antioxidant status was, however, similar for purple prairie clover with and without PEG [161], which suggests that the improved antioxidant status was not due to oligomeric and polymeric proanthocyandin. Antioxidant activity of proanthocyanidin fractions in vitro increased linearly up to fractions with mDP of 8–10, after which the activity levelled [162,163]. Therefore, proanthocyanidin and their building blocks might act directly or indirectly as antioxidants in the animal and might improve their health and product properties. 

## 13. Effect of Proanthocyanidin on Animal Performance and Animal Product Quality

Comparative feeding value in terms of sheep live-weight gain ranked perennial ryegrass < red clover < alfalfa < big trefoil < sainfoin < white clover in a summary by Ulyatt [164] and perennial ryegrass < red clover < alfalfa < big trefoil < birdsfoot trefoil < sulla < white clover in a summary by Waghorn et al. [165]. Comparative feeding value in terms of dairy cow milk solids (g/d; fat + protein) production ranked birdsfoot trefoil and white clover similarly, with both having higher feeding values than perennial ryegrass [165]. Therefore, the apparent feeding value of temperate proanthocyanidin-containing legumes is in general higher than non-proanthocyanidin (or trace)-containing perennial ryegrass, red clover and alfalfa, but similar to or lower than that of white clover. The high feeding value of white clover indicates that proanthocyanidins are not required for a high feeding value of legumes. The high feeding value of birdsfoot trefoil, big trefoil, sainfoin and sulla is therefore likely only partly explained by the presence of proanthocyanidin in their forge. Proanthocyanidin may, however, increase the feeding value as a result of improved energy efficiency due to reduced methane (energy) emission or reduced energy cost for urea synthesis, increased amino acid absorption in the small intestine, or improved overall animal health status. However, when the proanthocyanidin concentration in birdsfoot trefoil and big trefoil increase over 5% of DM, animal performance decreases due to decreased dry matter intake and/or excessively decreased digestion and availability of nutrients along the entire digestive tract [100,166]. Sainfoin and sulla, however, seem to be palatable forages which are preferred by ruminants even if they have a high proanthocyanidin level [167,168,169,170]. 

Ruminant products are high in saturated fatty acids (FAs), due to extensive microbial biohydrogenation of lipids in the rumen, which have been associated with health risks for human. Therefore, decreasing saturated FA and increasing unsaturated FA in animal products is desired. Feeding diets with proanthocyanidin have been found to decrease saturated FA proportion of lipids in meat and milk [171,172], likely due to inhibition of the biohydrogenation processes by microbes in the rumen [172,173]. However, the effect of proanthocyandin in the diet on milk and meat FA has been variable, likely dependent on the level and type of proanthocyanidin in the diet [171,173]. Feeding proanthocyanidin-containing forages was also found to reduce negative odour compounds in meat, like indole and skatole, which are normally high in meat from grazing sheep [174]. Indole and skatole are end-products of protein fermentation in the rumen. Therefore, the precipitation of dietary protein by proanthocyanidin and inhibition of proteolytic bacteria, as described above, are the likely mechanisms for the reduced indole and skatole formation. Reduction in indole and skatole formation were found to be greater at higher dietary proanthocyanidin concentrations [174].

Faeces of ruminants are the major source of *Escherichia coli* O157:H7, which can contaminate carcasses, and therefore meat, during slaughter. Ingestion of meat contaminated with *E. coli* can result in foodborne illness (food poisoning) in humans. Reducing *E. coli* O157:H7 shedding in faeces of ruminants will likely reduce meat contamination [175]. Phlorotannins from seaweed were found to inhibit *E. coli* in vitro [176] and in vivo [177] and to a greater extent than the proanthocyanidin from quebracho [176]. Proanthocyanidin from sainfoin had minimal effect on *E. coli* in vitro and in vivo [178], while proanthocyanidin from purple prairie clover reduced *E. coli* greatly both in vitro and in vivo [71,161,179]. The greater *E. coli*-reducing properties of proanthocyanidin from purple prairie clover than from sainfoin were thought to be due to the much greater PCC with both Rubisco and BSA, and increased outer membrane permeability and cell aggregation of *E. coli* due to purple prairie clover proanthocyanidin [71]. 

## 14. Summary

Proanthocyanidins from temperate/prairie forages bind preferentially with dietary proteins in the rumen, which can be disassociated in the acidic environment of the abomasum. This reduces the rate and extent of protein turnover in the rumen and may improve protein absorption in the small intestine and reduces N excretion into urine. The bioactivity of proanthocyanindins in forages to complex with dietary protein appears to be mainly related to their total concentration in the diet followed by molecular weight/mean degree of polymerization (increasing in activity up to 6–10 units) of the proanthycanidin. Dietary proanthocyanidin concentration should be sufficiently high (~>2% of DM in temperate forages) before positive effects can be detected, while too-high concentrations will impair feed digestion, intake (especially in *Lotus* species) and animal performance. Molecular makeup, orientation and bonds in the polymer chain appear to have little effect on the protein precipitating capacity of proanthocyanidin, but might be important in the binding strength in the protein complex and for their effect on microbes in the gut of the animal. Also, presence of high molecular weight proanthocyanidin fractions in feed, presence of gallated proanthocyanidin, and high protein precipitating capacity appear to be indicators for biological activity on gut microbes. Feeding mixed proanthocyanidins–flavonoids appears to function synergistically in increasing the biological activity of proanthocyanidin, at least against parasites in the gut. The high feeding value of proanthocyanidin-containing legumes could be the result of improved energy efficiency due to reduced methane (energy) emission, reduced energy cost for urea synthesis, increased amino acid absorption in the small intestine, or improved overall animal health status. The agronomic performance of these proanthocyanidin-containing legumes is, however, inferior to commonly used alfalfa, perennial ryegrass and white clover, which still prevents their uptake by farmers. 

## Figures and Tables

**Table 1:** Proanthocyanidin concentration, structure and protein precipitating capacity of several temperate forage legumes.

Trait	Legume Species						
	Sainfoin	Birdsfoot Trefoil	Big Trefoil	Sulla	Alfalfa	White Clover	Red Clover
	Forage						
Proanthocyanidin (g/kg DM) ^1^							
Extractable	44	7–36	61	35–84	0	ND	0.4
Protein-bound	38	9–13	14	9–31	0.5	ND	0.6
Fibre-bound	5	2–3	1	2–20	0	ND	0.7
Total	87	21–47	77	55–84	0.5	6–12	1.7
	Forage				Seed	Flower	
MW (DA) ^2^	2.0–5.1	1.8–4.4	2.2–3.9	-	3.6	-	-
mDP ^3^	4–12	6–14	8–44	3–46	5–7	10	9
Main polymer ^3^	Pdelph	Pcyanid	Pdelph	Pdelph	Pcyanid	Pdelph	Pcyanid
PD (%) ^3^	36–93	40–66	80–84	73–89	-	-	-
*Cis* (%) ^3^	47–88	84–85	76–88	69–84	-	-	-
Extender unit (%) ^3^							
Catechin	0	3–4	2–4	1–8	0	0	6
Epicatechin	11–27	27–67	13–19	9–18	92	0	81
Gallocatechin	7–19	5–7	6–16	14–23	0	39	6
Epigallocatechin	61–74	30–62	46–72	53–75	0	56	7
Terminal unit (%) ^3^							
Catechin	8–23	61–82	46–51	24–32	92	0	95
Epicatechin	22–47	16–21	13–20	0–6	0	0	5
Gallocatechin	18–40	2–17	20–16	50–66	0	48	0
Epigallocatechin	14–35	2–4	10–14	7–22	0	52	0
PCC (µg/mg) ^4^							
Alfalfa Rubisco	50	80	72	ND	108	ND	ND
Bovine serum albumin	269	436	323	ND	348	ND	ND

ND: not determined; ^1^ Values for sainfoin and birdsfoot trefoil from Scharenberg et al. [72], for birdsfoot trefoil and big trefoil from Terrill et al. [8], for birdsfoot trefoil, alfalfa and red clover from Jackson et al. [20], and for white clover from Burggraaf et al. [73]; ^2^ Molecular weight adapted from McAllister et al. [54], and Min et al. [74]; ^3^ Values for sainfoin from Koupai-Abyazani et al. [75], for birdsfoot trefoil from Foo et al. [65,76], for big trefoil from Foo et al. [65,77], for alfalfa seed coat from Koupai-Abyazani et al. [15], and for white and red clover from Sivakumaran et al. [18]. ^4^ Protein precipitating capacity (µg proanthocyanidin needed to precipitate 1 mg of alfalfa Rubisco protein or bovine serum albumin) adapted from McAllister et al. [54]. mDP: mean degree of polymerization; Pdelph: prodelphidinin; Pcyanid: procyanidin; PCC: protein precipitation capacity; MW: molecular weight; PD: prodelphinidin ratio.
